# Evaluation of mastoid volume and dimensions in unilateral microtia patients: retrospective study using High Resolution Computed Tomography (HRCT)

**DOI:** 10.1016/j.bjorl.2024.101501

**Published:** 2024-09-04

**Authors:** Trimartani Koento, Anita Amalia Sari, Mirta Hediyati Reksodiputro, Harim Priyono, Semiramis Zizlavsky, Reyhan Eddy Yunus, Joedo Prihartono, Mikhael Yosia

**Affiliations:** a Fakultas Kedokteran Universitas Indonesia, Departemen Ilmu Kesehatan Telinga Hidung Tenggorok – Bedah Kepala Leher, Jakarta, Indonesia; b Fakultas Kedokteran Universitas Indonesia, Departemen Radiologi dan Kedokteran Nuklir, Jakarta, Indonesia; c Departemen Ilmu Kesehatan Komunitas, Jakarta, Indonesia

**Keywords:** Volume, Mastoid, Microtia, HRCT mastoid

## Abstract

**Objective:**

The aim of this study is to evaluate mastoid volume and dimensions in patients with unilateral microtia using High-Resolution Computed Tomography (HRCT) to enhance the precision of reconstructive surgical planning.

**Methods:**

A retrospective analysis of HRCT mastoid scans from patients with unilateral microtia was carried out at Cipto Mangunkusumo General Hospital between May 2020 and August 2022. Parameters such as mastoid volume, height, and surface area were measured at the ear canal, Superior Semicircular Canal (SCC), and lateral SCC levels.

**Results:**

The analysis revealed statistically significant decreases in median mastoid air cell volume and mastoid bone volume in the affected ears compared to contralateral ears (*p* =  0.0312 and *p* =  0.02, respectively). Additionally, decreased mastoid height and surface areas at the ear canal and superior SCC levels were identified in affected ears (*p* <  0.05).

**Conclusions:**

Patients with unilateral microtia have diminished mastoid bone volumetric parameters and dimensions on the affected side. These findings offer critical data for surgeons in preoperative planning, enabling the selection of appropriate reconstructive techniques and providing comprehensive patient counselling.

**Level of evidence:**

Level 4.

## Highlights

• Reduced mastoid volumes in microtia ears offer novel anatomical insights.
• Microtia ears show smaller mastoid height and area at specific levels.
• Data aids in preoperative counselling and technique selection.
• HRCT valuable for precise mastoid anatomy evaluation in microtia.
• Mastoid variations crucial for optimal microtia surgical outcomes.

## Introduction

Microtia is a congenital disorder of the auricle that occurs with varying degrees of severity, from mild structural abnormalities to the absence of an auricle and middle ear strands. Microtia may be accompanied by ear canal atresia and conductive hearing loss. There is often a relationship between the degree of deformity of the auricle and the middle ear. Microtia is caused by developmental disorders in the embryonic stage, so there are often cases of microtia with disorders of the formation of the middle ear and other organs derived from the same embryological structure.^1^, ^2^, ^3^

The prevalence of microtia varies in each region, ranging from 0.83 to 17.4 per 10,000 births. The incidence of microtia is higher in Hispanic, Asian, and Native American populations.^1^ Men are affected more often than women, with a ratio of 2.5:1.^2^ The right ear is twice as commonly affected as the left ear (60%) and microtia is mostly unilateral (77% to 93%). This condition can occur independently or be related to several syndromes. The severity of microtia was classified into four categories by Marx in 1926.^2^, ^3^

The ear is an important organ that contributes to the aesthetics of the human face. The size, shape, position, and projection of the ear can be important components in auricle reconstruction, affecting a person's appearance. Additionally, microtia can cause psychological concerns related to the condition, such as the need for reconstruction surgery as well as possible hearing loss. Therefore, the purpose of reconstruction surgery includes two main goals: auditory and aesthetic functions.^4^

The abnormal growth and development of the ears can also lead to asymmetry of the body shape, including the facial area.^5^ A good projection of the ear after auricle reconstruction depends on the auriculocephalic angle and sulcus of the retroauricles. Conditions that can cause the auriculocephalic angle to shrink include rib absorption, contractures, height, and buttress height, potentially leading to unsatisfactory ear projections over time.^4^ Based on research conducted by Mayer et al., microtia is associated with aplasia or hypoplasia of the tympanic and mastoid processes of the temporal bone.^6^ In patients with microtia accompanied by facial asymmetry, mastoid, malar, and mandibular hypoplasia may occur. Abnormal side soft tissues, including skin, subcutaneous fat, and facial muscles, may be thinner than the normal side. The most prominent areas of deficiency are the temporal fossa, the malar region, and along the lower border of the mandible.^7^

The mastoid air cell system, which has a pyramidal shape, enlarges variably to all areas of the temporal bone. The pneumatization of the mastoid bone varies individually, and its development changes with age. The volume of mastoid cells can affect mastoid dimensions, which are assessed based on axial, sagittal, and coronal slices through computer tomography examination or High-Resolution Computed Tomography (HRCT) of the mastoid. Radiological evaluation using HRCT of the mastoid is important to assess the condition of the middle ear in microtia patients, which later plays a role in determining management if there is an abnormality.^8^, ^9^ Although mastoid air cells are interconnected, it is very difficult to measure volume directly. Therefore, a method is needed to accurately measure mastoid air cells. There is no technique that allows direct measurement of the mucosal surface area of the entire mastoid air cell system in vivo. The measurement of the volume of mastoid air cells using HRCT is easy to work with and provides highly accurate results. The axial cut of the temporal bone can represent the relationship between the tympanic and mastoid air cell system well. Anthropometry of mastoid area and height is easier to obtain on a CT scan.^10^, ^11^

Thus, the evaluation of mastoid volumetric parameters and dimensions in unilateral microtia patients is essential to obtain objective data, enhance patient education, and facilitate strategic planning for auricular reconstruction surgery.

## Methods

This study used an analytical comparison design of two paired groups (microtia ears with their contralateral sides) and was conducted using secondary data from HRCT mastoid exams. The study was carried out from May 2020 to August 2022 and was approved by the Ethical Committee of The Faculty of Medicine- Universitas Indonesia (protocol number: 22-09-1171), with secondary data confidentiality consent from the author. Inclusion criteria for the study were (1) Unilateral microtia patients at the Facial Plastic Reconstruction (FPR) Outpatient Clinic Cipto Mangunkusumo General Hospital, (2) Age 6–40 years, and (3) Had undergone HRCT mastoid examination at the PACS Department of Radiology at CMGH. Exclusion criteria included (1) Patients with a history of temporal bone trauma and (2) Patients with CT scan results showing damaged or temporal bone thickness <1 mm.

The secondary clinical data of unilateral microtia patients was obtained from the FPR division and matched with the CMGH Department of Radiology's Picture Archiving and Communication System (PACS) to identify the type of imaging that had been performed.

### HRCT mastoid

The HRCT mastoid scans were obtained from routine temporal bone images taken with patient in supine position without contrast nor sedation used in this procedure. The images were acquired using Philips Brilliance 64-slice CT scanner. All scans were obtained using the following parameters: tube voltage, 120 kV; effective mAs, 350; slice thickness, 0.6 mm; scan field of view, 180 mm; and image matrix, 512 × 512 mm. The images were taken in the axial plane. A coronal and 3D Multiplanar Reconstruction (MPR) were performed.^12^, ^13^

### Measurements

**Figure 1:**
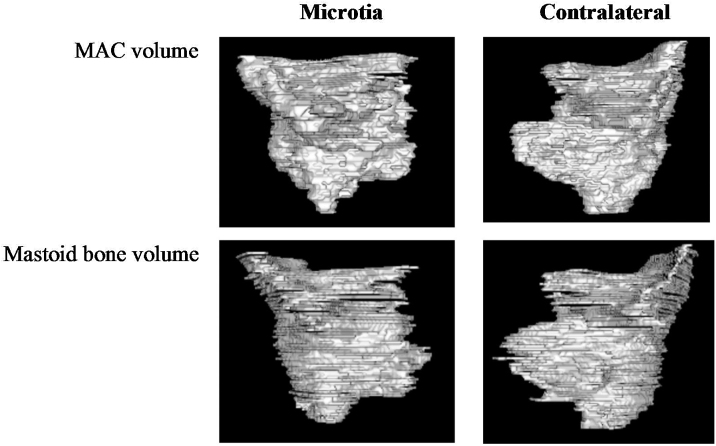
3D Reconstruction of mastoid volume in unilateral microtia.

**Figure 2:**
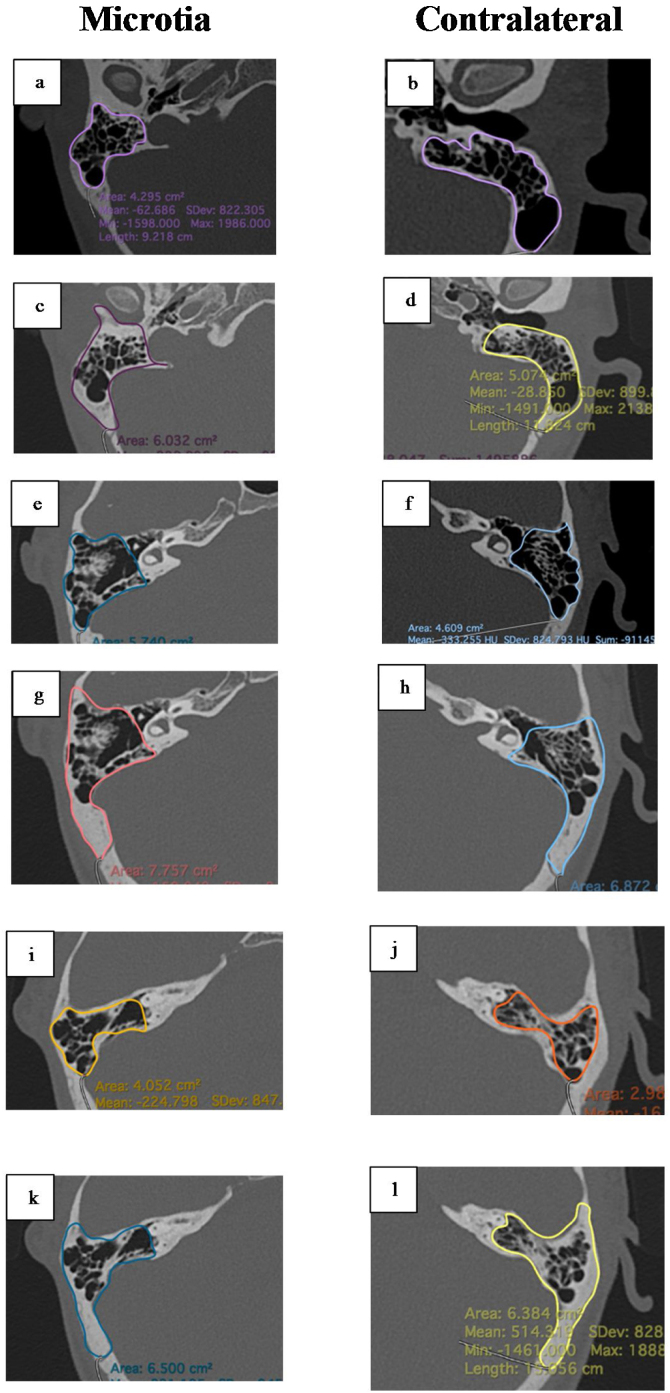
Measurement of MAC and mastoid bone are at various levels. Description: (a and b) MAC area at the ear canal level; (c and d) the area of the mastoid bone at the level of the ear canal; (e and f) MAC area at the lateral SCC level; (g and h) mastoid bone area at the lateral SCC level; (i and j) MAC area at superior SCC level; (k and l) mastoid bone area at the superior SCC level.

The mastoid area antropometric were consisted of volume and soft tissue thickness. The mastoid volume were measured in mastoid air cells without including the tympanic cavity with reference to aditus ad antrum based on axial plane of CT scan with 3D MPR technique and segmentation of each CT slice using the Horos software (Fig. 1). The mastoid height and surface area were evaluated at the the ear canal, superior SCC, and lateral SCC levels using axial plane HRCT scans of mastoid (Fig. 2). The HRCT mastoid data that was available in the Radiology Department's PACS system was re-read by a radiologist, and mastoid volume and dimensions were calculated using Horos software. Measurements were made by three different doctors who have more than 10 years of experience.

### Statistical analysis

All data were analysed using Statistical Package for Social Science (SPSS) version 20 statistical program. Data with a normal distribution were presented as mean and standard deviation, while data with an abnormal distribution were presented as median and range. Mastoid volumetric and dimensional assessments were analysed using paired t-tests when the data was normally distributed and Wilcoxon if the data was not normally distributed. A *p*-value < 0.05 was considered to be statistically significant.

## Results

### Subject characteristic

The number of microtia patients in the FPR outpatient clinic from May 2020 to August 2022 was 31, with 3 subjects having bilateral microtia and 28 subjects having unilateral microtia. Six subjects were excluded because they did not have complete HRCT data. All of the bilateral microtia subjects were female, while 23 of the unilateral microtia subjects were male and 5 were female. This study obtained 44 ears from 22 unilateral microtia subjects, which were divided into two groups: microtia and the contralateral side.

The distribution of characteristics was obtained from the 22 subjects involved in this study, 17 of whom were male and the remaining 5 were female. A total of 14 subjects were aged 6–18 years, and the remaining 8 subjects were aged 19–40 years. The median age of the subjects was 15.5 (8–28) years. The median body weight of the patients was 48 (20–90) kg, while the average height of the patients was 154 + 15.5 cm. The nutritional status of the patients was divided into 3 groups: 7 subjects were underweight, 7 subjects were of ideal weight, and the remaining 8 subjects were overweight, with a median value of Body Mass Index (BMI) of 20.5 (12.8–34.7). Fourteen subjects had 3rd degree microtia with right lateralization (Table 1).

**Table 1:** Subject distribution by characteristics (*n* = 22).

Subject characteristics	Freq	Percentage
Gender		
Male	17	77.3
Female	5	22.7
Age group		
6 18 y.o	14	63.6
19–40 y.o	8	36.4
Median (Min‒Max) age	15.5	8–28
Nutritional status		
Underweight	7	31.8
Ideal weight	7	31.8
Overweight/obesity	8	36.4
Median (Min‒Max) body weight	48.0	20–90
Average ± s.b height	154.0	±15.5
Median (Min‒Max) BMI	20.5	12.8–34.7
Lateralization		
Right	15	68.2
Left	7	31.8
Degrees of microtia		
1st degree	1	4.5
2nd degree	7	31.8
3rd degree	14	63.6

**Figure 3:**
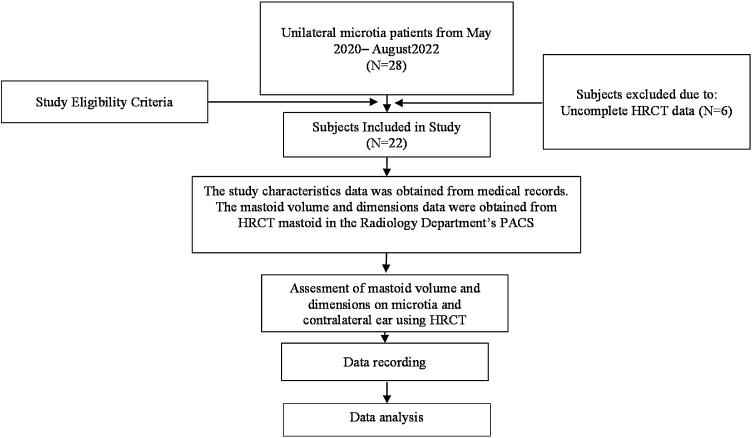
Study flowchart.

On the microtia side, the majority of the subjects had moderately severe conductive hearing loss or severe conductive hearing loss, while in the contralateral ear group the majority had normal hearing status. In the microtia group, 12 subjects had a Jarshdoerfer score above or equal to 7, while in the contralateral group all had a score above 7 (Fig. 3).

### Volumetric and dimensions assessment of unilateral microtia

The assessment of volume and dimensions of the mastoid in unilateral microtia showed that out of 9 parameters, 7 parameters were smaller on the microtia side, and 2 parameters were larger on the microtia side. Based on statistical analysis, 7 parameters were statistically significant, including mastoid air cell volume (*p* =  0.031), mastoid bone volume (*p* =  0.022), air cell area at the ear canal level (*p* = 0.031), bone area at the ear canal level (*p* =  0.000), air cell area at the superior SCC level (*p* =  0.006), bone area at the superior SCC level (*p* =  0.004), and mastoid height (*p* =  0.001). The results of the other parameters show that there is no significant difference between microtia and its contralateral side. The median volume of mastoid air cells in the microtia group is 6.49 (0.13–16.95) cm^3^, and the median of the contralateral group is 8.61 (1.28–17.61) cm^3^. The median volume of mastoid bones in the microtia group was 14.63 (6.39–33.04) cm^3^, and the median of the contralateral group was 14.99 (7.19–33.90) cm^3^ (Table 2).

**Table 2:** Mastoid volume and dimensions assessment based on HRCT.

Parameter	Microtia	Contralateral	*p-*value
	Mean (s.d))/Median (Min‒Max)	Mean (s.d)/Median (Min‒Max)	
MAC Volume (cm^3^)	6.49 (0.13–16.95)	8.61 (1.28–17.61)	0.031^a^
Mastoid Bone Volume (cm^3^)	14.63 (6.39–33.04)	14.99 (7.19–33.90)	0.022^a^
MAC SA Level Ear Canal (cm^2^)	2.66 (0.44–5.56)	3.14 (0.71–4.37)	0.031^a^
MAC SA Level Lateral SCC (cm^2^)	3.25 (0.49–7.16)	4.11(0.80–6.23)	0.263
MAC SA Level Superior SCC (cm^2^)	3.14 (0.49–6.77)	4.41(0.58–7.95)	0.006^a^
Mastoid Bone SA Level Ear Canal (cm^2^)	5.30 (1.54)	4.56 (1.32)	0.000^a^
Mastoid Bone SA Lateral SCC (cm^2^)	6.03 (1.91)	5.89 (1.50)	0.431
Mastoid Bone SA Level Posterior SCC (cm^2^)	5.52 (2.34)	6.41(1.84)	0.004^a^
Mastoid Height (cm)	2.98 (0.67)	3.20 (0.55)	0.001^a^

MAC, Mastoid Air Cell; SA, Surface Area.

a Significant *p*-value *p* <  0.05.

## Discussion

In this current study 77.3% subjects were male. Jovic et al.^14^ reported that 64% of microtia patients were male. The degrees of microtia are 3rd degree (63.6%) with lateralization on the right ear (68.2%). González-Andrade et al.^15^ and Suutarla et al.,^16^ reported about 60% of unilateral microtia patients had a right lateralization. Widodo et al.^17^ in her research had majority of Grade 3 in the case of microtia. In the microtia group, the majority of hearing loss was moderately severe and severe conductive hearing loss. This is in accordance with the research conducted by Suutarla et al. and Ishimoto et al. the majority of microtia patients have conductive hearing loss on the affected side of the ear.^16^, ^18^ Majority of subjects had a Jarshdoerfer score above or equal to 7 and all contralateral sides had a normal Jarshdoerfer score. The results of the research of Ishimoto et al. showed no significant association between hearing loss and a total CT scan score as assessed by the Jarhsdoerfer scoring system.^18^

In this study, both MAC volume and mastoid bone volume showed a smaller median volume on the microtia side, with statistically significant differences. Measurements were carried out using the segmentation method to obtain accurate results. To date, no studies have been published on the calculation of MAC and mastoid bones in unilateral microtia patients. However, several studies have reported mastoid pneumatization in microtia patients. For example, Mayer et al. reported reduced mastoid pneumatization on the microtia side with aplasia or hypoplasia of the tympanic segment or temporal bone mastoid process.^6^ Takes et al. also reported hypopneumatization or stunted development of mastoids on 57.1% of microtia sides, and Gautam et al. reported that 71.4% of microtia sides do not have mastoid air cells or have reduced ones.^8^, ^19^ The temporal bone petrosa segment is underdeveloped in patients with MFD. Mastoids are a complex structure, and the presence of chronic inflammation of the middle ear can cause impaired middle ear ventilation and poor gas exchange, which may lead to a reduction in mastoid pneumatization on the microtia side. Based on this study, larger mastoid bone sizes in the contralateral group may indicate the presence of temporal bone growth inhibition on the microtia side.

The main purpose of ear elevation surgery in microtia reconstruction is to achieve sufficient and long-lasting projection of the reconstructed auricle, with results comparable to those of a normal ear. Several factors can affect post-reconstructive ear elevation, including post-wound contractures, wound healing, and hemifacial microsomia. The height of the costal cartilage implanted into the posterior surface of the reconstructed auricle may also have an influence on ear elevation. Additionally, the small mastoid volume in microtia patients can affect the temporoauricular angle, which may, in turn, affect the projection of the ear in Stage 2 of auriculoplasty.^4^

Other parameters measured in this study were mastoid area at the level of the superior SCC, lateral SCC, and ear canal, as well as mastoid height. The area was divided into measurements of the area of air cells and bones. At the level of the superior SCC, both MAC and bone surface area were larger on the contralateral side and statistically significant (*p* <  0.05). At the ear canal level, larger sizes were obtained on the microtia side and were statistically significant (*p* <  0.05). At the lateral SCC level, the area on the microtia side was smaller in the air cell calculation, but larger in the bone calculation. The existence of these differences and variations shows that mastoid volume measurement should be carried out by segmentation on each slice, not just based on a certain level. The larger size of the mastoid area in the ear canal area was caused by the fact that there is no ear canal on the microtia side, so the area was replaced with mastoid bone, which caused the size at that level to be larger. The height of the mastoid on the microtia side was lower than on the contralateral side (*p* < 0.05).

A limitation of this study is the small sample size and that the measurements in this study are approximate and depend on the ability of the research team to measure accurately. Even though repeated measurements have been taken by three different measuring people, the possibility of human error still exists.

## Conclusion

According to the findings of this study, the MAC and mastoid bone volume of the microtia side were significantly smaller in unilateral microtia. Furthermore, other parameters such as mastoid surface area and height were also diminished in comparison. These observations imply a potential for unfavourable auricular projection after microtia reconstruction, which could be attributed to the differences in mastoid anatomy size in unilateral microtia. The implications of these results are substantial, as they may enable surgeons to provide patients with more informed preoperative counselling and to select the appropriate technique for auricular reconstruction surgery. However, further prospective research utilizing High-Resolution Computed Tomography (HRCT) of the mastoid is required to evaluate and compare mastoid volume and dimensions while considering the patient's physical condition.

## Authors’ contributions

Trimartani Koento: Principal investigator, conceptualization, patient recruitment.

Anita Amalia Sari: Plastic reconstructive surgery consultation and procedures.

Mirta Hediyati Reksodiputro: Study design, manuscript revision, senior oversight.

Harim Priyono: Otorhinolaryngology expertise, clinical evaluation.

Semiramis Zizlavsky: Data acquisition, senior Otology specialization.

Reyhan Eddy Yunus: Radiological expertise, patient management.

Joedo Prihartono: Statistical analysis, data interpretation.

Mikhael Yosia: Manuscript preparation, literature review.

## Funding

This study was funded by International Indexed Publication Grant (PUTI) Q2 Fiscal Year 2022–2023 (Batch 3) from the Ministry of Education, Culture, Research, and Technology, Indonesia. Grant Number: NKB-1406/UN2.RST/HKP.05.00/2022.

## Conflicts of interest

The authors declare no conflicts of interest.
